# Report on the El Paso Mass Casualty Incident Hospital Response: Enhancing Surge Capacity

**DOI:** 10.5811/westjem.24991

**Published:** 2025-08-29

**Authors:** Susan F. McLean, Nancy Weber, Adam Adler, Alejandro Rios Tovar, Stephen Flaherty, Alan H. Tyroch

**Affiliations:** * Texas Tech University Health Sciences Center El Paso, Department of Surgery, El Paso, Texas; † Texas Tech University Health Sciences Center El Paso, Department of Emergency Medicine, El Paso, Texas; ‡ Texas Tech University Health Sciences Center El Paso, Department of Orthopedic Surgery, El Paso, Texas; § University of Texas Health Rio Grande Valley, Department of Surgery, Edinburg, Texas; ** Del Sol Medical Center, Department of Trauma and Surgical Critical Care, El Paso, Texas

## Abstract

**Introduction:**

On August 3, 2019, a mass casualty incident (MCI)/active shooter event in El Paso, TX, left 21 people dead on scene and 27 transported. Our main objective in this article was to describe trauma center responses to a sudden patient influx after a MCI/active shooter event. We hypothesized that a triage practice in which two physicians providing care while simultaneously triaging would be equivalent to triage with a single physician providing triage only. The secondary objective was to describe patient injuries and treatment. Our third objective was to describe how a large, multidisciplinary team of hospital personnel were rapidly notified and arrived at the trauma center. Finally, we describe how the problems identified in a review of hospital response led to better results after implementing new practices in a 2023 MCI/active shooter event.

**Methods:**

We conducted a retrospective cohort/medical record review and departmental survey. We performed the Fisher exact test using survival as an outcome to compare the two centers’ triage methods.

**Results:**

A total of 15 patients arrived at the University Medical Center of El Paso, 14 of them within 35 minutes and one in a later transfer; 14 survived at 24 hours. Total patients included 10 females and 5 males, mean age 40.6 (1–88) years. Mean hospital length of stay (LOS) was 13 ± 16.4 days. For the six intensive care unit (ICU) patients the mean LOS was 5.7 (1–11) days. In comparing day 1 survival between the center where a surgeon and an emergency physician triaged patients while also providing care and the center where a sole triage physician was on duty, survival rates were equivalent (*P* = .56). Six surgeries occurred on day 1 with four laparotomies performed within 43 minutes. The trauma team expanded rapidly as 132 persons from 16 departments, notified by phone calls and text, arrived, improving communication and patient registration in two 2023 MCI responses.

**Conclusion:**

The survival rate of victims of a mass casualty incident brought to a Level I trauma center and triaged by a surgeon and an emergency physician who simultaneously provided care did not differ significantly from the survival rate at a Level II trauma center with a single triage physician on duty. The rapid arrival of multiple specialists resulted in 14 patients treated within an hour.

## INTRODUCTION

On August 3, 2019, at 10:39 am, an active shooter instigated a mass casualty incident (MCI) in El Paso, Texas. This shooting left 22 people dead in the first 24 hours and sent 27 to local hospitals immediately.^1^ This event was the second of three active shooter events in the United States that week.^2^ The incidence of MCI active shooter events, defined as a wounding event with > 3 casualties,^3^ increased throughout the early 2000s: >160 MCIs have occurred since 2000 with over 1,000 casualties.^3^ According to a Department of Justice report written in a collaboration with the Federal Bureau of Investigation and Texas State University, MCI/active shooter frequency has increased from one incident recorded in 2000 to an average of 16.4/year in the US.^3^ Thus, trauma centers continue to evolve their responses to these events. Hospital response is frequently reported per specialty.^4^–^6^ In-hospital response from triage to operating room (OR) is especially important.^7^–^9^ Most reviews focus on triage and surgical and emergency department (ED) response,^5^ with others delineating specialty response.^10^ Analysis of a hospital response in a Level 1 trauma center could assist planning for future MCI/active shooter events.

Our objective in this review was to characterize the in-hospital response of a Level I trauma center— University Medical Center (UMC) of El Paso—to a MCI/active shooter event. We hypothesized that using a triage system in which two triage physicians simultaneously treated patients would have a survival rate similar to that in a triage system in which there was a sole, designated triage physician who did not also provide care. Our secondary objective was to characterize patient injuries and treatments. We also sought to characterize the rapid influx of hospital staff and the methods used to notify them that the MCI was occurring. Finally, we describe the problems identified in reviewing the hospital response to the MCI and new practices implemented in a MCI/active shooter event four years later in 2023, and in a citywide mock MCI that same year, which resulted in improvements in responses.

## METHODS

Our institution’s Investigational Review Board for Human Subjects Research approved this study. We conducted a retrospective cohort medical record review of only those patients arriving at the trauma center who had sustained wounds in the MCI/active shooter event; we also analyzed the performance of the Hospital Incident Command System (HICS). We used the Federal Bureau of Investigation definition of MCI/active shooter: an incident in which at least three persons are killed during a shooting event 3 Data sources were medical records and HICS data from the HICS incident commander at the Level I trauma center, who was also the trauma program manager. We also obtained data from three other hospitals: a Level II trauma center, Del Sol Medical Center, and two Level III trauma centers. Data was emailed to us by the trauma medical director at the Level II center and by the trauma program managers at the two Level III trauma centers. We obtained additional data on numbers of patients and overall survival rate at the Level II center.

We obtained data from the Level I trauma center in two ways: via survey and by medical record review. We surveyed department heads regarding how personnel were notified and the number who arrived at the hospital. This information was used to inform the section on expansion of the trauma team and personnel deployment. Clinical data was obtained from medical records of patients identified to have a gunshot wound in the MCI/active shooter event. A separate team was created in medical records for patients injured in the event. We obtained all information regarding time to OR, and types of injuries, operations, and procedures from the medical records. We calculated surgical timing from ED arrival times and OR perioperative nursing documentation. Time to OR was calculated as arrival time to surgery start time. We obtained information regarding hospital administrative response from an after-report from the MCI/active shooter event, and an after-report from a mock MCI four years later. Two authors also were present during the subsequent MCI/active shooter event in February 2023 and recorded information concurrent with the incident. Data regarding total numbers of patients in the August 2019 MCI were obtained from the HICS commander. Information about the total numbers of patients in the 2023 MCI/active shooter event were obtained from news reports and the HICS commander. We were only able to access Injury Severity Score (ISS) data from the Level I trauma center. The Fisher exact test was used for categorical variable comparison (survival statistics).

**Population Health Research Capsule**

What do we already know about this issue?

*The number of mass casualty incidents (MCI)/active shooters has increased in the past 20 years in the US*.

What was the research question?

*Is a triage practice in which two physicians triage patients while simultaneously providing care equivalent to a single physician providing triage?*

What was the major finding of the study?

*The two triage practices had equivalent survival rates (P = .56)*.

How does this improve population health?

*This comparison of triage practices and a summary of institutional response to a MCI may be helpful to hospital disaster planning committees*.

The results of improvements made to the system after 2019 are reported in two incidents: the first in a February 15, 2023, MCI/active shooter event at a local shopping mall, and the second at a citywide, mock MCI drill in October 2023. We obtained this information through direct observation and via news reports.^11^ The news reports were used to verify dates and numbers of victims at the scene. We obtained data regarding the citywide mock MCI from a report at the Level I trauma center.^12^ In addition, two authors (SFM and AHT) participated in the mock MCI.

## RESULTS

### Transport and Early Timeline

**Figure 1:**
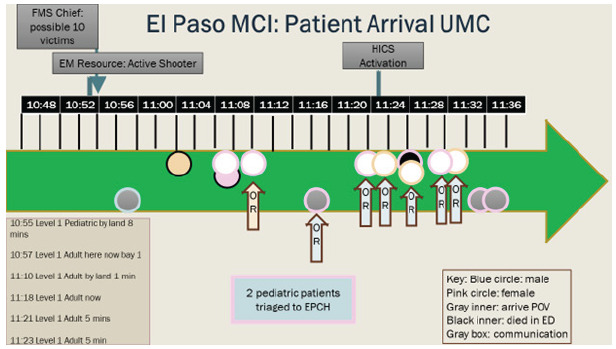
Timeline of patient arrival and Hospital Incident Command System activation at UMC. *Note: FMS, The El Paso Fire Department and Emergency Medical Services work in combination. *MCI*, mass casualty incident; *UMC*, University Medical Center; *EMS*, emergency medical services; *HICS*, Hospital Incident Command System; *EM*, emergency medicine; *EPCH*, El Paso Children’s Hospital; *POV*, privately owned vehicle; *ED, e*mergency department.

The initial 9-1-1 call occurred at 10:39 on August 3. 2019, followed by EMResource notification at 10:53. (EMResouce is a notification system to multiple clinicians in the region). By 10:54, an emergency medical services (EMS) captain notified UMC of multiple casualties. A surge of 14 victims arrived at the Level I trauma center within 34 minutes. Ten of these patients were Level 1, the highest level of trauma activation, and four arrived by private vehicle (Figure 1). Eleven patients arrived at the Level II trauma center, and two Level 3 patients were transported to two Level III trauma centers. Transport from scene was rapid: the Level I trauma center is 4 miles and the Level II center is 2.5 miles from the MCI scene. We were unable to obtain more specific triage, injuries, ISS, and death-at-scene data as this information was withheld for use in the criminal proceedings. In addition, we only had access to medical records or patient information from the Level I trauma center.

The Regional Area Council (RAC) for Trauma Service Area I supplied an ambulance bus, but it was not used.

### Outcomes

At UMC, the mean ISS was 9.9 ±11.5 (1–38) of the 10 female and 5 male victims. Fourteen survived. Of the victims brought in the first hour, one patient arrived without vitals, underwent an ED thoracotomy, and died of injuries sustained during the shooting. Another patient arrived later that day as a transfer, thus totaling 13 patients who survived the first surge. A final patient arrived in transfer later, for a total of 14 survivors. Mean age was 40.6 ± 22.6 years (1–88 years). Mean hospital length of stay (LOS) was 13 ± 16.4 days. Six patients went to the intensive care unit (ICU): mean ICU LOS was 5.7 ± 4.1 days (2–11); hospital LOS for these six was 22.6 ± 19.6 (10–62.) Median ventilator days for three patients was eight days. The one death occurred at UMC due to a thoracic vessel wound that was discovered after resuscitative thoracotomy. Of the 11 patients brought to the Level II center, there were 9 survivors after 24 hours. The two deaths at that center were due to one patient experiencing an acute myocardial infarction during resuscitation and one elderly patient who received comfort care only and subsequently died. An additional death occurred at the Level II center months later.

### Triage

**Figure 2:**
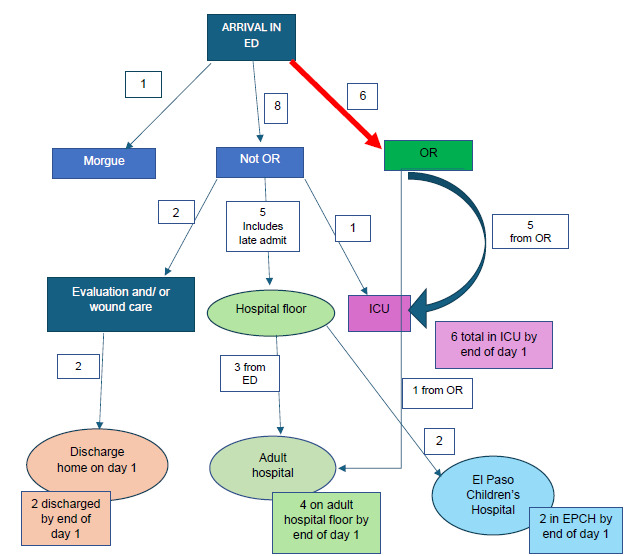
In-Hospital Triage of shooting victims at Level 1 Trauma Center *ED*, emergency department; *EPCH*, El Paso Children’s Hospital; *OR*, operating room; *ICU*, intensive care unit.

At the Level I trauma center the first activation came at 10:57, and the first patients arrived at 11:03. Because the MCI/AS occurred on a Saturday when few clinicians were present, it became necessary to both triage and care for patients at the same time. In prior mock MCI events patients arrived presorted with colored tags attached, but in the aftermath of the shooting we had to treat patients without tags and without a second physician to perform triage. In contrast, the Level II center had sufficient staff, which allowed for one physician to focus solely on triaging patients. Five of the initial 14 patients arrived with a level 1 activation (L1A). All patients would have been designated L1A if each had arrived outside the setting of an MCI, but some were not activated as such due to arrival by privately owned vehicle (Timeline, Figure 1). If a patient with normal vital signs arrived but had been shot, these patients were seen by emergency physicians and general surgeons; it wasn’t necessary to activate L1A because surgeons were already present in the ED, as were the radiology, laboratory, and respiratory therapy services. Neither was a patient sesignated LIA if they were initially seen in the hall, thereby bypassing the trauma bays and going straight to the OR. When the first patient arrived, the surgical trauma team on site included one general surgeon trauma attending, one fifth-year surgical resident, two second-year surgical residents, two interns, and an orthopedic team that consisted of one faculty attending and two residents. The ED had two faculty attendings present. Anesthesia had one faculty and two nurse anesthetists (CRNA) present. The ED attending and one general surgeon performed triage and patient care simultaneously. A general surgery attending and a surgical resident and an ED attending and an emergency medicine resident were in each of 12 trauma rooms. At triage the patients were sent to the OR, to a floor in the main hospital or children’s hospital, or to the ICU after imaging studies. No tagging systems were used (Figure 2). The disposition of 15 patients, including one later arrival, was as follows: four to regular floor; two to home from the ED; one directly to the ICU; five to ORs; one to the morgue; and two to an adjoining pediatric hospital. By the end of the day, five of the six patients who underwent surgery went to the ICU, and one to the regular floor (Figure 2). The overall triage system at the Level I trauma center functioned well because initially there were empty ORs, and the ICU space had been expanded into an empty pre-op area.

Our main objective in this study was to review the in-hospital response at our Level I trauma center; we received additional information from the Level II center and the two Level III centers. At the Level I trauma center, we had one general surgeon and one orthopedic surgery attending present at MCI onset. The Level I center subsequently called in eight additional attending surgeons (six general, one orthopedic, and one cardiac) totaling 10 attending surgeons. The Level II center had two general surgeons on duty and called in seven additional attending surgeons. Because the Level I center already had one general surgery attending on call, that surgeon did simultaneous rapid triage with the ED attending, as well as performing initial surgical treatment. In contrast, at the Level II center two general surgeons were on duty, including the trauma medical director, who served as triage officer and performed no initial surgeries. That center called a trauma surgeon over from a nearby Level III center, as well as orthopedic and neurosurgeons, and an additional general surgeon who had privileges at the Level II center.

At our Level I trauma center, 14 of 15 patients survived—a 93% survival rate at 24 hours. Of the eleven arrivals at the Level II center nine survived, for 82% survival at 24 hours. We documented no significant difference in percentage survival (*P* = .56) between the Level II triage system, which had a separate triage physician, and the Level I center where one general surgeon and one emergency physician simultaneously triaged and treated patients.

### Trauma Teams Personnel Surge: General Surgery and Orthopedic Surgery

The entire general surgery department was activated including subspecialists in pediatric, endocrine, oncology, colorectal, and cardiothoracic surgery. The subspecialists arrived 30–45 minutes after MCI activation, assisting with line placement and evaluations for Advanced Trauma Life Support. The cardiovascular surgeon assisted orthopedic surgery in one case involving an extremity. The first two orthopedic surgeons operated on an open thigh wound; and when that patient needed repeat surgery, a cardiovascular surgeon joined this team to stop the bleeding. This same patient had an exploratory laparotomy concurrent with orthopedic surgery. Among the procedures performed to stabilize fractures and treat orthopedic injuries using damage-control methods were the following: external fixation; irrigation; splinting; and debridement (Tables 1 and 2). Operative triage began at UMC with the first group; a patient had a trans-abdominal gunshot wound and went to the OR from the ED without occupying a trauma bay, with one general surgeon in the hospital and one chief resident arriving within 15 minutes of patient arrival. Four primary laparotomies were performed on day 1; one additional laparotomy was returned to the OR for bleeding within an hour (Table 1). Twenty-six surgeries were performed on the subsequent 2 through 191 days after the MCI. The mean initial laparotomy start time from arrival was 25.7 minutes. General surgery, orthopedic, and bedside procedures are listed in Table 2. Procedures performed later are listed in Table 3. The second procedure was an ED thoracotomy by a second general surgeon who arrived from outside the hospital. With his arrival two trauma surgeons were on site. The patient who had a thoracotomy performed in the ED died, thus freeing the second general surgeon to perform laparotomies. Two postgraduate year-5 and numerous lower-level residents arrived within 20 minutes. The second and third laparotomies occurred concurrently.

The first general surgeon who performed laparotomy 1, returned to the ED and assessed a new patient with indications for surgery. That patient needed both laparotomy for a trans-abdominal gunshot wound (GSW) and left thigh exploration for GSW to left thigh. This first general surgeon, freed up after laparotomy 1, then performed laparotomy 3 and adjunctive procedures concurrent with two orthopedic surgeons exploring an extensive bleed from a left thigh wound. This patient returned to the OR from the post-anesthesia care unit (PACU) within an hour after the thigh restarted bleeding; the cardiovascular surgeon assisted orthopedic surgery with femoral vein ligation. The second general surgeon, who was available after the ED thoracotomy, operated on a patient with multiple GSWs and a transpelvic GSW, laparotomy 3. A third general surgeon who arrived during these procedures took the fourth laparotomy to the OR. This fourth laparotomy was completed rapidly. After the fourth laparotomy patient entered the PACU, the patient had to return to the OR within an hour for bleeding. This bleeding required repeat laparotomy for hemorrhage control. A fourth general surgeon and a pediatric surgeon arrived at laparotomy 3 after the laparotomy had been performed, and they placed an additional central line. All four laparotomies were damage-control procedures. A fifth general surgeon (colon and rectal) arrived after the laparotomies had been done in the OR and started compiling a list of patients from the MCI/active shooter event. A sixth general surgeon arrived and attended to a patient in the ED who did not require surgery on day 1.

The surgeons had face-to-face communication in the PACU and later communicated via cell phone. The deployment of arriving attending surgeons was coordinated verbally, by surgeons checking in and reporting back to the ED and PACU. Surgical residents and emergency physicians worked in the trauma rooms to identify patients who likely needed laparotomy or orthopedic procedures, thereby enhancing patient flow from the ED to the OR The adjacent locations of the ED/OR/PACU also helped patient flow.

In summary, there were four primary laparotomies, one thigh exploration for the initial cases, and one ED thoracotomy. These cases required three general surgeons and two orthopedic surgeons who were present or who arrived within 30 minutes. The fourth general surgeon placed a central line on laparotomy patient 3. One patient returned to the OR for laparotomy for bleeding, and one returned for thigh bleeding. These cases occurred in the first 90 minutes after arrival of 14 patients. There were two additional orthopedic surgeries on day 1.

### Trauma Team Surge at University Medical Center

To delineate total hospital staff response, we surveyed 16 departments for numbers and methods of call-in (Table 4). A total of 132 additional personnel arrived from the 16 departments surveyed. Mean number of employees called in per department was 8.3 ± 7.1; mean 94.4% ± 25.5%. Notification methods included the following: text messaging, 12 departments (75%); phone call, 14 departments (87%); phone tree, three departments (19%); and pager, two departments (13%). Eight departments (50%) reported self-arrival (without specific call-in). Two departments used four methods of contact, four departments used three methods, seven departments used two methods, and one department used one method of contact.

### Adjuncts to Personnel Surge

#### Transfusions and Settings

A massive transfusion protocol aided in transfusion of 49 units of packed red blood cells (PRBC). Six patients received a mean of 8 PRBC, 6.4 fresh frozen plasma units (FFP), 12 platelets, and 8 × 10 pack units cryoprecipitate. The hospital settings themselves enhanced care for the trauma patients: nearly empty ORs on a Saturday, and ICU bed expansion into the empty pre-op area.

#### Problems with Mass Casualty Incident response at Level I trauma center, UMC, and Early Solutions

The first problem was limited information from the scene, which was resolved by trauma bay reports from EMS. A second problem was that the hospital switchboard became overwhelmed by calls from the community. A third problem—double registration with “trauma names” and real names—was resolved during physician rounding. A fourth problem was delayed HICS activation, which did not occur until 30 minutes post-patient arrival. The delayed HICS activation impelled healthcare workers responding to use texts and phone calls to alert other practitioners that a MCI/active shooter event was occurring and to report to the hospital.

To address problems, the hospital disaster committee reviewed the HICS incident. To resolve registration confusion, the hospital adopted the Texas Wristband Project, in which a patient receives a wristband with a number that stays with the patient throughout the trauma episode, from incident scene through hospital admission. To prevent the hospital switchboard from being overwhelmed, the Regional Area Council for Trauma now designates multiple places around the city for family reunification centers. To assist with medical personnel notification, the Level I trauma center, as well as Texas Tech University Health Sciences Center at El Paso, have instituted emergency alert systems that go out over pager, cell phone, and email. To aid documentation, the ED faculty created a short emergency history and physical exam form.

The impact of these changes has been tested twice since 2019. A shooting incident with four victims occurred at the Cielo Vista Mall in El Paso on February 15, 2023. Three victims survived, and one died.^11^ Two patients were transferred to the Level I trauma center. The emergency notification mobilized > 50 physicians and nurses to the trauma center. Both victims survived. A mock MCI, modeled as a multiple plane crash with 100 victims, was staged on October 12, 2023, at the El Paso International Airport (EPIA) as the triennial citywide emergency exercise. Nineteen total patients including two pediatric patients were sent to the Level I trauma center at UMC (the center we report on here).

Improvements were seen in hospital and personnel activation (Table 5). Hospital activation occurred early, at the start of the incident. The HICS activation at the Level I trauma center occurred prior to the first patient leaving the scene: > an hour earlier than in the August 3, 2019, MCI. All patients had a numbered wristband and so were identifiable from scene to hospital. There were no duplicated entries. Personnel activation improved: Hospital notification occurred at 8:45 am; HICS personnel activation to group A (departmental leaders) occurred at 09:33 am; first patient left scene at 09:40 am; and a second HICS activation occurred at 10:28 am. At 11:03 am messaging advised of a meeting in the Level I trauma center boardroom for debriefing.^12^. These times for entire hospital activation were quicker than during the August 3, 2019, MCI. Thus, over one subsequent MCI/active shooter event and one mock MCI in 2023, the changes made after the initial August 2019 incident sped up hospital response and clarified patient identification from scene.

## DISCUSSION

A Level I trauma center in El Paso, TX, responded to a rapid patient influx after an MCI/active shooter event. Practices that facilitated response were a simplified triage method, timely arrival of trauma team and associates, and immediate ICU expansion. Medical practices enhancing hospital response included damage-control surgery and massive transfusion policy. All patients who arrived with vital signs survived.

### Triage: In Hospital

Triage is essential to differentiate patients requiring immediate surgery from patients requiring workup and disposition. There are two issues with expanding trauma teams in response to an MCI. One is personnel availability, and the second is patient arrival times. When there are few personnel to handle a rapid patient influx, triage and care must be simultaneous, involving both emergency physicians and general surgeons. Two other hospitals in the US specifically used a combined triage with EM and trauma surgery after an MCI: 1) Beth Israel Deaconess Medical Center after the Boston Marathon bombing in Massachusetts (April 2013); and Scott & White Memorial Hospital during the Fort Hood, TX, MCI/active shooter event (November 2009), where an ambulance-bay triage team composed of emergency physicians and one general surgeon organized initial care.^7^,^13^ Hirschberg et al modeled trauma response in a computer simulation based on actual MCIs. He noted that up to 4.6 critical patients per hour could be treated with in-house trauma teams, but that with expansion an increase to 7.4 critical patients per hour was feasible.^14^ In El Paso, the Level I trauma center had one general surgeon available at the start; hence, initial triage was provided by the this general surgeon, the emergency physician, and associated residents. As in the computer model, bringing in more trauma team members increases the ability to care for more patients.

Having the emergency physician and general surgeon perform both triage and patient care is advantageous for treating patients when there are short patient-arrival times. With longer arrival times, more personnel can be redeployed from other areas or from home to increase trauma team size. During the El Paso MCI, the receiving hospitals had <10 minutes from initial notification until patient arrival. Similarly, during the Boston Marathon bombing, the five receiving hospitals were within two miles of the MCI scene, which led to short response times.^6^,^13^ Hospital responses for the Virginia Tech MCI/active shooter event in Blacksburg, VA (April 2007), and the Fort Hood MCI were longer at 23 minutes and >one hour to patient arrival, respectively.^4^,^7^ Thus, reported patient arrival times after MCI activation has ranged from 10 minutes to >one hour. While both the Level I and II trauma centers receiving patients in El Paso had short patient arrival times, the two hospitals had equivalent survival rates. Having a general surgeon and emergency physician to treat victims while simultaneously triaging was associated with equivalent survival rates to having separate triage officers.

Triage for severity may be inaccurate during an MCI. It is reported that only 10–15% of patients arriving during an MCI are critically injured.^14^ In over-triage the limited resources of a trauma team are depleted on patients who are less severely injured than initially expected; conversely, under-triage leads to the most severely injured patients not getting rapid care. Perfect triage is difficult: a retrospective review of two MCIs in Israel noted that even experienced trauma surgeons initially classified only 7 of 15 “severe” injuries as severe, (ie, undertriaged) and classified three mild-moderate injuries as “severe” (ie, over-triaged).^15^ A retrospective review of a binary triage that was used prehospital in the terrorist attacks in Paris, France, in November 2015 noted a 36% under-triage and 8% over-triage rate.^16^ Similarly, a prospective study of MCI in-hospital triage demonstrated 24% over-triage and 16% under-triage.^17^

These three articles demonstrated varying rates of over- and under-triage in MCI events. It is critical to use resources efficiently in an MCI; hence, the presence of an emergency physician and a general surgeon at UMC to simultaneously triage and treat likely avoided delays in care from waiting until trauma personnel arrived from outside the hospital. In summary, when patient arrival times are fast, it is imperative for the trauma teams present to both triage and continue care, as happened at our Level I trauma center during the MCI/active shooter event in 2019. Using the emergency physician and the general surgeon on duty to both triage and provide care was not associated with increased mortality.

### Turnover by Damage Control

The Level I trauma center surgeons used the principles of damage control surgery (DCS) to preserve patient physiology as well as turn over trauma teams. In trauma surgery DCS refers to not completing the surgery but instead immediately treating life-threatening injuries.^18^ Advantages of DCS include the ability to correct physiological derangements before definitive surgery. Both orthopedic and general surgeons used DCS.^19^ Trauma surgeons used abbreviated laparotomy with temporary closure, and orthopedic surgeons used external fixation and washout. In trauma cases requiring > 3 transfusions per hour, DCS has been associated with increased survival after laparotomy.^19^ The use of DCS was key in other civilian traumatic MCIs: in Paris in 2015; in 12 civilian mass shootings in 2016; and for orthopedic injury management during the 2015 Amtrak derailment in Philadelphia, PA.^10^, ^20^, ^21^

A second reason for using DCS, besides physiology restoration, is that using DCS further helped turn over staff and ORs. Remick et al discussed that in military MCIs, surgeons need to maintain situational awareness of battles, the probability of further injuries, and resource utilization.^9^ This military DCS or “mDCS” combines physiological and clinical variables that prompt usual damage-control decisions in surgery (clinical DCS [cDCS”]) with combat variables that contribute to ongoing injuries). When civilian trauma teams used situational awareness to treat patients in a civilian MCI, combined with typical reasons for DCS patient physiological characteristics, this has been termed mass casualty DCS (mcDCS).^9^ In the El Paso MCI the initial laparotomies met criteria for cDCS due to hypotensive, acidotic, or blood loss criteria for DCS.^10^, ^20^, ^21^, ^23^ In addition, the use of DCS, which was clinically indicated, also aided patient throughput from ED to OR to PACU and, thus, could be considered “mcDCS.”

The practice of early DCS, correction of blood volume, and having multiple teams perform surgeries has been reported in both military and civilian MCIs.^24^,^20^ In Paris, at the Bégin Military Teaching Hospital, five ORs were used for performing 24 surgeries in 24 hours.^20^ The four laparotomies at our Level I trauma center in El Paso occurred with an average time to OR of 25 minutes; comparably, in a review of the Paris 2015 terrorist attacks, it was found that average time to OR for the most injured was 147 minutes.^16^ In the El Paso MCI/active shooter event, DCS aided both transport to OR, if needed, and rapid re-deployment of surgeons and staff for further trauma care.

### Transfusion

Massive transfusion protocols were used for six patients. In both the 2013 *Prospective, Observational, Multicenter, Major Trauma Transfusion (PROMMTT)* study and the 2015 PROPPR (Pragmatic, Randomized Optimal Platelet and Plasma Ratios) clinical trial, the importance of maintaining a transfusion ratio of 1:1:1:1 of FFP: PRBC:platelets:cryoprecipitate was demonstrated to decrease day one mortality from hemorrhage.^25^, ^26^ The Level I trauma center had in place a massive transfusion protocol that specified the specific blood product components brought up to the ED and OR. This specification included products that, if given in order, would give the correct ratio of FFP:PRBC:platelets: cryoprecipitate to adhere to a 1:1:1:1 ratio. The massive transfusion protocol enforced adherence to the 1:1:1:1 transfusion ratio at our Level I trauma center has been reviewed and published elsewhere. 27

### Team

Rapid expansion of all personnel departments was integral to the MCI response at the Level I trauma center. In simulation studies, increasing team numbers was associated with ability to care for more patients.^14^ Reports on the Boston Marathon bombing were similar to the El Paso MCI situation; the bombing occurred on a holiday, meaning there with fewer planned surgeries in the hospitals, and many staff arrived quickly as they lived nearby.^6^,^13^ At our Level I trauma center, the MCI occurred on a Saturday; hence, while the ORs were mostly empty, a smaller cohort of healthcare workers was present on the weekend, meaning staff did have to come in to the hospital. This might have caused a delay; however, all the patients who had surgery survived. In contrast, during the Fort Hood MCI, sufficient surgical staff were already present.^7^ At our Level I center, there was rapid expansion of other hospital staff in addition to surgeons, which was critically important for trauma management: environmental services for OR/ED room turnover; and nursing staff in the OR, ED, and ICU. Per an after-action survey, a total of 132 personnel arrived.

### Mass Casualty Incident Problem Response

Problems in our Level I trauma center response included communications and registration, which were comparable to what has been previously reported. Often receiving hospitals did not have accurate information from the scene.^4^,^7^ During the Fort Hood MCI, it was noted that the Level I center did not receive reports on intrahospital transfers until helicopter contact due to phone line obstruction. The sending hospitals suffered from switchboard jamming at the receiving hospital after the number was publicized.^7^ During the Virginia Tech MCI, there was a reported lack of communication from the scene to the hospital.^4^ Another issue is that short notification prior to arrival^6^,^10^ occurs when hospitals are close to the scene.

The second issue with communication/electronic medical record was patient identification and registration, including dual name registrations per patient, a problem solved during physician rounds and by in-person identification. During the Boston MCI, identification problems at Beth Israel Deaconess Hospital were solved by creating a special MCI team.^13^ The Texas Wristband Project, which introduced a scene-number wristband, will likely solve identification issues. The wristband project was initiated prior to 2019 and was piloted by nine Regional Area Councils for Trauma and Emergency Services from December 1, 2020–January 31, 2021. The project came to fruition approximately a year and a half after the 2019 MCI/active shooter event. The wristband project was put into place by Texas Senate Bill 500 and the Department of State Health Services in Texas to keep track of patients being transferred by EMS in large-scale disasters. Using the wristband, a patient will have one alpha-numeric identifier throughout his course of care from scene to whichever hospital he is transferred. This identifies the patient, who will keep the the same identifier throughout the course of care.^28^

## LIMITATIONS

This retrospective cohort study may have been subject to recall and reporting bias. The study is limited by legal aspects of the 2019 MCI including ongoing legal actions, which prevented precise description of ballistics, weapons, and cause of death on scene. Additionally, a retrospective study depends on health-record and time-reporting accuracy. An additional limitation was that the comparison cohort in this study was a nearby hospital that had a single, separate triage clinician on duty. Thus, there was not an identical patient comparison group, leaving the possibility of selection bias; our Level I trauma center may have had injuries more amenable to successful treatment. It should also be noted that we had limited access to patient medical records at the Level II center; therefore, those patients’ injuries are incompletely described. Neither was ISS data available to compare the Level II center to our Level I center. Thus, we were unable to compare patient severity.

We used the outcome “survival” when comparing the two triage systems. It is probable that survival was determined by specific injuries and their severity, and not necessarily by the triage system itself. Further, because it was not possible to capture data from all hospitals (eg, if a patient tripped and fell and sustained injury during the MCI, and then presented to the Level II or III centers and was not transferred to our Level I center, we would not have captured that patient as an injury related to the MCI/active shooter event. Regarding the trauma team expansion, the survey data was compiled based on the recall of chiefs of service and could, therefore, have been affected by recall bias. The timing of surgeries was based on times reported from ED admission to the OR. Admission to the ED was listed as the time a patient was registered; however, because so many patients arrived in a short time, the registrar may have registered some at the same time, for convenience. Finally, at both the Level I and Level II centers, both groups had small numbers, increasing the risk of a type II error.

## CONCLUSION

The seventh most deadly active shooter event in US history occurred in El Paso, TX, on August 3, 2019.^2^ We describe the hospital response at our Level I trauma center. In characterizing this response, multiple factors including rapid influx of hospital personnel, a massive transfusion protocol, and the use of damage- control surgery practices were likely helpful. These factors were not compared to other hospitals in the area since this data was not collected and not part of this study. Our Level I center had a small number of surgeons and emergency physicians initially to receive the injured patients. By necessity, the triage officers simultaneously provided care. Noting later that at the Level II center the trauma team had a separate triage officer, we hypothesized that survival rates between the two centers would be comparable. And, in fact, the two triage systems were associated with no significant difference in survival at the end of the first day between the two centers. Thus, we conclude that when a trauma center is pressed to provide initial triage and trauma care rapidly in a mass casualty incident/active shooter event of this size, a small group of clinicians can initiate trauma care and triage simultaneously.

## Figures and Tables

**Table 1:** Initial six surgeries.

Case	Time to OR hrs/min	1^st^ OR case	2^nd^ OR case (time to OR since end of first case)
1.	00:01	Exploratory laparotomy Hepatic packing Evacuate duodenal hematoma Pack back and flank wounds AbThera placement	NA
2.	00:36	Exploratory laparotomy Small intestinal resection, multiple sigmoid resection Bilateral tube thoracostomy AbThera placement	NA
3.	00:23	Left anterior resuscitative thoracotomy Exploratory laparotomy Splenectomy Small intestinal resection AbThera placement	Re-exploratory laparotomy Sigmoid colectomy Diaphragm repair Ligation IMA AbThera placement (00:08)
4.	00:43	Left chest tube Exploratory laparotomy, hepatic packing Explore and ligate bleeding vessels left thigh Right femoral CVP, right femoral arterial line Left subclavian CVP Pack wounds	Re-explore left thigh and ligate left femoral vein (01:40)
5.	4:12	Debride left open humerus fracture, External fixation left open humerus fracture	
6.	4:48	Bilateral thigh irrigation and debridement Open arthrotomy and irrigation and debridement right knee	

*AbThera*, temporary abdominal closure system; *OR*, operating room; *hrs/min, hours/minutes*; *NA*, no answer; *IMA*, inferior mesenteric artery; *CVP*, central venous pressure.

**Table 2:** Bedside procedures.

Case	Day	Procedures	Comments
1	1	Irrigation and splinting right index finger	Discharge day 2 after MRI brain
2	1	Irrigation wound and excision bullet right gluteal cleft	Discharged from ED
4	1	Right lower extremity bedside incision and drainage	
5	1	Right thigh wound care	Went to wound care 8 weeks post discharge from ED on day 1
6	1	Right tube thoracostomy Left tube thoracostomy eFAST ultrasound Echocardiogram	Transfer to ICU
7	3	Right internal jugular CVP	
10	3	Echocardiogram	
10	19	EGD/PEG	
	52	EGD and pancreatic stent removal	
11	1	Irrigation and debridement left thigh wounds	
13	1	ED resuscitative thoracotomy, right tibial IO catheter	
14	3	Right internal jugular CVP	Frequent blood draws, patient had MI

*MRI*, magnetic resonance imaging; *ED*, emergency department; *ICU*, intensive care unit; *eFAST*, extended focused assessment with sonography in trauma; *CVP*, central venous pressure; *EGD*, esophagogastroduodenoscopy; *PEG*, percutaneous endoscopic gastrostomy, *IO*, intraosseous; *MI*, myocardial infarction.

**Table 3:** Subsequent surgeries.

Case	Number of days since arrival	Case description
3	4	Right elbow fracture irrigation and debridement, Integra and wound vac placement
	37	Right elbow irrigation and debridement and split thickness skin graft 6 × 4 cm
8	4	Irrigation and debridement, exploration, open reduction and internal fixation of distal and proximal phalanx and rotational flap of right thumb
	10	Right breast reconstruction with oncoplastic technique using an inferiorly based glandular cutaneous flap. Left mastopexy for symmetry
9	1	Re-exploration of open abdomen, abdominal fascial closure Debridement and washout right back and flank wounds and removal foreign body × 2 Left thigh medial and lateral wound irrigation, debridement and wound vac placement, primary closure of lateral wound
	5	Irrigation and debridement left leg wound, exploration femoral vessels, irrigation and debridement right medial leg wound, replace packing right flank wound
	25	Irrigation and debridement left thigh wound after dehiscence with removal of skin ellipse 5 × 3 cm.
10	2	Re-exploratory laparotomy, left hemicolectomy, small bowel resection with anastomosis, transverse colostomy, traumatic diaphragmatic hernia repair, gastrorrhaphy, left abdominal wall component separation and abdominal wall closure
	2	Cystoscopy, bilateral retrograde pyelogram, left ureteral stent placement
	13	Incision and drainage abdominal wound and left upper back wound
	29	Midline wound washout and placement of ACE ll wound matrix 7 × 10 × 2 cm and ACELL powder for enterocutaneous fistula; placement negative pressure wound therapy device
	46	Isolation of enterocutaneous fistula with 24 Fr Foley catheter; split-thickness skin graft 187 cm squared
	54	Cystoscopy and left ureteral stent removal
	191	Colostomy reversal, enterocutaneous fistula resection, adhesiolysis, PEG removal, abdominal wall reconstruction with bilateral component separation
11	19	Excision of left buttock foreign body
12	2	Re-exploratory laparotomy, small bowel resection and anastomosis, segmental transverse colectomy with end colostomy, Moss feeding tube placement, proctoscopy, right thigh debridement, left tibia washout and removal foreign body Right hand irrigation debridement and splinting
	5	Exploratory laparotomy, repair of traumatic left iliac hernia, right lower extremity irrigation and debridement, right chest tube placement
	6	Irrigation and debridement right hand gunshot wound, wound exploration, foreign body removal × 2.
	10	Operative screw fixation of left anterior column acetabulum 6. Right hand gunshot wound irrigation, debridement, removal of foreign body fragments
	13	Irrigation and debridement left gluteal gunshot wound for seroma
	46	Irrigation and debridement and metacarpal fracture joint replacement with a Pyrocarbon implant
	153	Colostomy reversal, proctoscopy, scar revision; right hand foreign body removal, left gluteus irrigation and debridement and foreign body removal.
14	4	Irrigation and debridement, open reduction and internal fixation of left Humerus shaft fracture
	83	Removal of left antecubital fossa foreign body
15	1	Re-exploratory laparotomy, abdominal closure, debridement of right flank wound

*PEG*, percutaneous endoscopic gastrostomy: wound vac, vacuum-assisted closure; cm, centimeter.

**Table 4:** Hospital personnel arriving for response to mass casualty incident.

Department	Number of employees who arrived	Percentage of department on site	Comments
Surgery Staff plus residents	20	50	Persons on call or backup were called automatically. Text messaging alerted department. 100% of those called came, if they were in town.
ED Staff and residents	16	39	This number includes staff already in hospital.
ED Mid-levels	4	54	ED mid-levels called plus those already present. Two later shift mid-levels arrived early.
Anesthesia Staff and CRNA Staff	16	43	4 in house, 12 came; 8 called in and 3–4 self-arrived.
Orthopedic Surgery Staff plus residents	18	50	50% called and 50% came in; some responded to texts.
OR nursing	20	95	95% of department present.
ED nursing	8	50	Combined, all people who came in would be ≈ 50% above average staffing
ICU nursing	15	15	15% total ICU nursing came in when not scheduled, 13% showed up and 2% called.
Radiology techs	6	25	50% of department called, 50% of those came in, for 25% additional staff overall.
Radiology staff	5	50	100% of called came in, 50% department in hospital.
CT techs	3	19	100% of those called came in.
Environmental services	8	6	100% of those called came in.
Blood bank	3	26	Second-shift employees called in early; supervisor saw post on Facebook and self-arrived.
Nutrition Services	12	15	3 called in, and 9 stayed over shift time to make 12 total respondents.
Guest Services	12	19	100% of those called came in; 7 called and 5 additional came in.

*CRNA*, certified registered nurse anesthetist; *ED*, emergency department; *OR*, operating room; *ICU*, intensive care unit, *CT*, computed tomography.

**Table 5:** Table of comparisons for the three mass casualty incident activations.

Date	Number patients Received	First notice To L1TC	First patient arrival	HICS active	HICS debrief	HICS deactivated	Family Meeting Center	Problems/Comments
08/03/2019	14^1^	10:53	11:05	11:20	13:05	15:00	13:17 MacArthur Elementary School.	Late HICS activation Communication with public Family meeting area not publicized until late in day, leading to persons coming to hospital and crowding in ICU waiting area, which is near entry point. Switchboard jamming at L1TC Patients had dual registrations, causing clinicianss to take hours to clarify.
02/15/2023	2^2^	17:24	17:27	0^3^	NA	NA	18:20 Est. at Burgess High School	No HICS activation Crowding due to staff who were not providing treatment lingering in ED treatment areas.
10/12/2023	17 2 sent to EPCH, affiliated with UMC, the L1TC	08:45	9:50^4^	09:33	11:03	12:12	Mock drill, no center established by office of emergency management or RAC.	AOD asked the hospital operators to activate MCI; however. operators correctly activated HICS both overhead and on pagers/texts. Triage area in ambulance bay was crowded; however, clinicians were well dispersed. No impediments to patient care. Registration used Texas Wristband numbers, improved.

1 During event, at scene 4 injured, 1 dead on scene, 2 transported to UMC or L1TC, and 1 patient sent elsewhere.

2 4 total victims, 1 dead on scene. 2 patients transported to UMC the L1TC.

3 HICS not activated. The L1TC security and police were activated. RMOC activated at 17:27 and deactivated at 21:34. Both patients admitted to UMC, the L1TC. HICS not activated due to smaller patient load but was noted to be a problem that could have corrective action, as there were problems with crowding. Emergency communications did not go out to associates.

This incident was a citywide mock massive casualty event, the EPIA Triennial Exercise. In this mock scenario a plane crashed at the airport. There were 100 victims, 72 sent to area hospitals. In the After-Action Report and Improvement Plan, first patient transported off scene from the EPIA at 09:40 am per EMResource. (EMResouce is a notification system to multiple clinicians in the region). The fastest that a patient might have arrived was at 11:40 am, although this was not recorded in the after-action report. The HICS activation was timely. Healthcare staff were in place for first patient arrival. Previous registration problems were resolved by using wristbands. With the Texas Wristband Project, trauma patients are issued a numbered wristband that stays with them through all encounter—ED, admission and even transfer to another facility. However, registrars noted that this number had to be entered into three places.

*UMC*, University Medical Center; *L1TC*, Level I trauma center; *HICS*, Hospital Incident Command Center; *EPIA*, El Paso International Airport; *RMOC*, Regional Medical Operations Center; *RAC*, Regional Advisory Council for Trauma, the BorderRAC.
